# An investigation of transportation logistics strategy on manufacturing supply chain responsiveness in developing countries: the mediating role of delivery reliability and delivery speed

**DOI:** 10.1016/j.heliyon.2022.e11283

**Published:** 2022-10-27

**Authors:** Moh’d Anwer AL-Shboul

**Affiliations:** Business Administration Department, King Talal School of Business Technology, Princess Sumaya University for Technology (PSUT), P.O. Box 1438 Al-Jubaiha, Amman, Jordan

**Keywords:** Transportation logistics, Strategy, Delivery reliability, Delivery speed, Supply chain, Responsiveness, Manufacturing firms, Middle east

## Abstract

This study aims to investigate and examine the impact of delivery reliability (DR) and delivery speed (DS) on the relationship between a manufacturing firm's transportation logistics strategy (TLS) and supply chain responsiveness (SCR). Furthermore, it examine the impacts of SCR on manufacturing firm performance (MFP). A quantitative methodology was used for the purposes of gathering and analysing primary data for this empirical study, which included 212 participants in large-sized manufacturing firms in the Middle East Territory. A web-based survey was used for the data gathering process and applied after conducting a small pilot study. The conceptual model was tested by using a hypothesis-testing deductive approach. The findings are based on covariance-based analysis and structural equation modeling (SEM) using AMOS software. The findings show that DR is mediating partially the relationship between TLS and MSCR, and that DS is mediating fully the relationship; further, it is pointed out that SCR is supported with improved MFP. The empirical findings can have insightful implications for managers and practitioners in terms of boosting competitive advantage and financial performance.

## 1 Introduction

Due to globalization and volatile economy, most products life cycles become shorter and reduce quickly and continuously, customers' demand and their requirements are changing quickly and rapidly, leading most firms to decrease the lead-time and be more responsive. From this perspective, firms who are able to respond quickly to the changes in such working in a dynamic environment will be the primary determinant for its performance. This can only be achieved when the performance of the entire supply chain (SC) is reliable, responsive and resilient. Therefore, most manufacturing supply chains are expected to react quickly and effectively to the changes that firms face even it divers internally of externally (Praharsi et al., 2022; Asamoah et al., 2021). Thus, the manufacturing firm and its strategy must manage its internal (transportation logistics operational competence) and external (market competitiveness) effectively and efficiently to be more responsive to the requirements of the local, regional and global markets. Indeed, the production and shipping of products and services on time without any delay, to the right location, in right quantity, and at minimum total cost is one of the crucial aims for many manufacturing firms due to the recent trend of the global challenges (Diaz et al., 2022; Sahoo, 2021). Considering the increased highlighting on sustainability within the SCM discipline, it is somewhat surprising that the focus on transport logistics and its strategy within manufacturing firms is relatively limited and scarce in the SCM literature (Eriksson et al., 2022). Hence, there is a need a contribution from scholars and practitioners to analyse transport and its strategy within manufacturing firms as a part of wider logistics and SCM contexts McKinnon (2021). Therefore, this study comes to investigate the role of delivery reliability (DR) and delivery speed (DS) on the relationship between a manufacturing firm’s transportation logistics strategy (TLS) and supply chain responsiveness (SCR). Furthermore, investigate the impact of SCR on manufacturing firms’ performance (MFP).

Overall, supply chain responsiveness refers to how channel members successfully react in a cooperative fashion in order to deal with environmental changes (Nenavani and Jain, 2022; Praharsi et al., 2022; AlHusian and Khorramshahgol; 2018). Yang et al., 2019a, Yang et al., 2019b argue that it helps companies to use successfully use their skills to ward off adverse impacts Giannakis et al. (2019) of turbulent environments and better react to these changes. Backstrand and Fredriksson (2020) argue that it permits firms to respond in persist way and an adequate period to the demand of clients in order to maintain their competitive advantage. In this regard, the adoption of supply chain responsiveness can lead transportation logistics in companies to achieve their goals in an efficient way. In fact, Kottala and Herbert (2019) report that when companies change from sea to railways truck to airways, the speed of transport can increase. Sharma and Modgil (2019) reveal the importance of logistics and the vulnerability of supply chains to adverse events have bolstered managers to support supply chain responsiveness. Salehzadeh et al. (2020) report that whole supply chain and logistics enhancement lead to better service and customer satisfaction through trade liberalization, the formation of multinational logistics alliances, and networks.

Many borderless firms have been forced into the modern business environment as a result of intense competition to improve the performance of their processes, operations, and different logistical activities (i.e. transportation, etc.) through re-evaluating and re-examining their SCP not only to ensure the needs of customers for their products, but also focusing on the speed of access, and rapid response to their desires (Nenavani and Jain, 2022; Md. Rafiqul et al., 2020). There are some requirements and standards that have become prerequisites for many manufacturing firms and their SC network to remain responsive and competitive in light of the global race in dynamic markets to enhance their profitability and raise the level of performance such as time to market, flexibility, short-lead times, fit to order fulfilment customers, minimizing order returns (accuracy), and optimizing costs (Backstrand and Fredriksson, 2020; Salehzadeh et al., 2020; Xu et al*.*, 2017).

Most firms are currently moving towards increasing their global competitiveness through their enjoyment of flexibility and high dynamism and responsiveness to any changes that might be happening in the markets. Thus, most of their supply chains and transportation logistics have become a potential benefit to achieve a competitive advantage to improve their performance and serve their customers in a perfect way to let them be more satisfied. However, the current intensified competition is not exclusively between different manufacturing firms, but also among other competitors and partners external to their SC network (Aljanabi and Ghafour, 2021; Sharma and Modgil, 2019).

Using and implementing different transportation modes, capacity, operations, and several SC logistical activities in order to achieve and enhance the firm's performance via its SC (Eriksson et al., 2022; Andersson et al., 2019). Hence, there is one way to reduce the negative environmental impact and costs of transport is to change the mix of transport modes applies such as using an intermodal transport solutions (Reis and Macario, 2019). Therefore, SC is responsible for all physical movements and holding storage of items, raw materials, components, semi-finished goods, and items from the supplying point to the consumption point. The key purpose of effective transportation operations in any firm in any country is to enhance the focal firm’s SCR (Huge-Brodin et al., 2020; Ning et al., 2019a, Ning et al., 2019b). Thus, not all firms have appropriate responsiveness in spite of they have and use different transportation modes. Many Personal Computer producers such as Gateway and Compaq have failed in delivering their products to customers on time due to failure to react quickly to provide the required items and parts for manufacturing PCs from their providers in Taiwan (Jazairy et al., 2021; Chen et al., 2018; Qrunfleh and Tarafdar, 2013). What is needed to bridge the gap? The question now is, what do we need to fill the gap between TLS and SCR to customers’ orders, wants, and needs? Responsiveness in SC is successfully achieved by preparing and establishing appropriate strategic elements that support logistics transportation operations at any firm. Furthermore, to do this is through using different transportation mode(s) that accomplish certain firm’s criteria (i.e. on-time delivery, etc.) such as highly dependable and reliable mode(s) with high flexibility and speed (McKinnon, 2021; Ning et al*.*, 2020).

Regarding the main context of this paper, the literature mentioned that still there are some points to answer. Firstly, studies examine SCR and TLS separately to avoid the influence of one factor on the others; while, literature has considerable details on SCR (Asamoah et al., 2021; Barrot et al., 2020). In addition, the effects associated with the adoption and/or using different reliable and flexible transportation mode(s) are not conceptually indicated (Giannakis et al., 2019). Similarly, there are several research studies that consider SCR a significant factor to enhance and improve a firm's performance (Nenavani and Jain, 2022; Asamoah et al., 2021; Heinen et al., 2019). Whereas, many pieces of research in SCM literature did not consider the dependable and flexible delivery modes to be deployed in conjunction with firms’ TLS and SCR, and the benefits as well. Secondly, no clear findings on the effect of TLS on SCR on firm's performance (Eriksson et al., 2022) The Tsvetkova and Gammelgaard (2018) and Qi et al. (2009) studies show that there is a significant and positive impact between firm’s TLS and SCR. While, other studies show that TLS do not include directly of SCR, but they desired to use it as mediating factor to do so (Reardon et al., 2020; Yang et al., 2019a, Yang et al., 2019b). Finally, the literature reveals that does not offer enough insight on how the “gap” between the main appropriate TLS that should be offered at any firm and SCR are bridged. However, a lack of research theoretically demonstrates how this should be applied (McKinnon, 2021).

By addressing the previous gaps, this research will address several issues as follows: (1) investigate and identify the appropriate TLS at any manufacturing firm that supports SCR, (2) examine and analyse the effect of moderating factors of different delivery characteristic modes (i.e., delivery reliability and delivery speed) on the linkage between manufacturing firm’s TLS and SCR. The researchers also focus on the effect of SCR on MFP.

SCR is a crucial indicator of how TLS at any MF and its operations fulfil its goals since it indicates the capability of SCR and its strategy to adapt to the frequently changing consumers' behaviour for their needs and preferences and ultimately lead to enhanced firms' performance, and its SC as well (Yang et al., 2019a, Yang et al., 2019b; Aggrwal, 2018; Qrunfleh and Tarafdar, 2013). For this study, the researchers have developed a conceptual research model and empirically tested and validated it by proposing two logistical transportation elements, delivery reliability, and delivery speed, respectively, mediating the relationship between TLS and SCR. This is in addition to the researchers’ proposition that SCR leads to enhancing and improving the firm's performance. Therefore, this study contributes to completing the framework of the literary SC theories in shedding light and giving a conceptual overview that takes into account the two logistical transportation criteria of delivery reliability; and delivery speed as mediating parameters for improving SCR from supporting main necessary and appropriate TLS. The findings of this research explain the significance of implementing such delivery transportation modes criteria that support the main TLS elements. Furthermore, it indicates that applying more SCR will enhance the performance of manufacturing firms.

The research structure is orderly as follows: section 2 explains the background of the SCM literature review in the discipline of a strategic view of TLS; SCR and firm’s performance. Section 3 demonstrates the conceptual research framework and hypotheses. The research methodology will be presented in Section 4, while section 5 illustrates the discussion of findings and theoretical contributions. Finally, the researchers present managerial implications, limitations of the study followed by suggested directions for upcoming research work, and a conclusion.

## 2 Theoretical background related literature review

In the global markets, and during the last decade, most of the firms are facing many different challenges due to the rapid development of technology as the product life cycle has become shorter as well as the changing market demands (Jha et al., 2022; Yang et al., 2019a, Yang et al., 2019b). Therefore, many manufacturing firms and their several logistical activities in general, logistical transportation operations in particular, are increasingly driven by clients wants rather than forecasts (Giannakis et al., 2019). Sahoo (2021) argue that recently most SCs have become more volatile, unpredictable, and increased product propagation has made offering it on-time without delay is very critical in order to avoid obsolete inventories; further, manufacturing firms' strategies are concerned about how they should be able to develop their transportation logistics by offering several modes in their industry relative to its competitors. Therefore, the firm's quick response is considered an essential parameter in determining its level of performance, and success in the market (Diaz et al., 2022; Pinheiro et al., 2020). If the manufacturing firm has the ability and responding quickly to demands in their unstable and uncertain environment; the advantages include increased flexibility, in operations, improved speed in delivery, shortened throughput cycle time, shortened lead time and declined cost, improved quality of services, enhanced product innovativeness, and superior competitive advantage (Praharsi et al., 2022; Giannakis et al., 2019). Therefore, responsiveness enables firms to quickly detect the market change signals, share information among other partners/firms, and reengineer their internal processes to meet new market requirements, adopt and implement new technological processes ahead to be more competitive among other competitors (Asamoah et al., 2021; AlHusain and Khorramshahgol, 2018). Therefore, the balance between efficiency and responsiveness depends mainly on the types of products and services offered by firms, functional type products should adopt efficient SC strategy, whereas, innovative type products should implement responsive SC strategy (Seclen-Luna et al., 2021). Thus, it is significant to recognize some factors that may consider critical and play a crucial role in the firm's ability to quickly, effectively responds to the environmental changes, and lets firms be more competitive and flexible (Reis and Macario, 2019).

SCR is defined as a system, and how much the system has a speed to set its output within the obtainable domain of four external flexibility types as product, mix, volume, and delivery, in response to external factors, i.e. inventory replenishment. Further, the ability of SC to respond to market demand in a time-effective manner (Chetty et al., 2020; Giannkis et al., 2019; Ning et al., 2019a, Ning et al., 2019b). SCR is the ability of all partners within the SC is able to react purposefully and within an appropriate time-scale manner (time frame, schedule etc.) to customer orders and/or changes in the market place, to create or sustain competitive advantage (Narayanan and Shekar, 2020). In this situation, responsiveness is considered one of the most important practices needed to be implemented by manufacturing firms to attain a relative advantage (Ayoub and Abdallah 2019; Banerjee et al., 2018). Aljanabi and Ghafour (2021) state that by increasing the demanding levels of customization and product variety in the marketplace, manufacturing firms with the capability to react quickly to meet customer demands on time will achieve an important and decisive competitive advantage over their competitors. Towards sub-sectors of the manufacturing industry electronics/IT, food processing, chemical/cosmetics, textile, pharmaceutical, and furniture, firms are formulating their strategies in order to enhance their responsiveness to customer demands by providing various products with very short lead times through suitable and convenient delivery mode(s). Thus, SCR became a vital factor of competitive advantage for several firms (Diaz and Golenbock, 2022; Aggarwal, 2018).

Due to the dynamic and intensified competitive environment, several manufacturing firms now call to conduct assessments to be more responsive for completing its SC activities (convert basic commodity from upstream into a finished product in the downstream) (Praharsi et al., 2022). Consequently, well-integrated SC is one of the essential actions for logistical transportation strategies to improve SCR (Tsvetkova and Gammelgaard, 2018). A practical SCR assessment tool should be able to quantify both the efficiency and effectiveness of SC action (Hale et al., 2020; Yang et al., 2019a, Yang et al., 2019b). Seclen-Luna et al. (2021), indicate that SCR was tested based on three prime factors: reliability, cost, and time (respond). Since, these factors consider the outcome of SC operations in addition to that its ability to let's offer all required customers' needs be available with their hands at the least cost and fast, at the right location, without delay, and in good condition (McKinnon, 2021).

A number of firms have made many improvements that have reduced and mitigated deficiencies due to poor supplier response, unexpected customer requirements, and needs in an uncertain business environment (Jha and Sharma, 2022; Feng et al., 2019). Therefore, the recognition and focus on the importance and role of SCR as a phenomenon have brought wide concern among corporate directors, practitioners, and scholars in conquering the business universe. Whereas, the rapid and continuous changes in the world of knowledge, globalization, and rapid intense competition between various firms, especially industrial ones, have forced most firms to adapt and adopt the SCR feature in an attempt by these firms to obtain a competitive advantage and stay in the circle and orbit of competition (Sahoo, 2021; Ning et al., 2019a, Ning et al., 2019b; Shi et al., 2019).

However, the form and pattern of the traditional production and distribution operations has changed in a pivotal and radical way, which has forced most firms to focus on the importance of re-design their manufacturing and distribution network in many different countries around the world (Ning et al., 2020). Therefore, many firms faced several emerging challenges, especially in warehousing and logistical transportation, such as arriving at the place on time, obtaining the required product quantities on time and at the lowest cost, and with the correct invoices. Thus, during the last decade, firms began to emphasize that currently competition is not only between firms, and is not enough to improve their performance and efficiency, but they should also focus on the importance of achieving the advantage of their SCR (Andersson et al., 2019; Qrunfleh and Tarafdar, 2013).

Seclen-Luna et al. (2021) reveal the existence of different kinds of responsiveness in terms of the time horizon concerned (e.g. medium or short-terms responsiveness) and the unit of change (e.g. volume and delivery responsiveness). They also profess that a supply chain can show various levels of responsiveness which depends on where in the supply chai nits responsiveness is measured. Jha et al. (2022) tried to investigate the performance of efficient supply chain management end how to enhance the performance of the current supply chain with the aim of developing a model of a responsive supply chain. They reveal that such a model can be used as a Mirmohammadi et al. (2017) attempt to develop an efficient model for optimization of the logistics and supply chain systems including transport and environment conditions. That is why they develop a linear mathematical programming model for the green vehicle routing problem with time windows and time-dependent urban traffic (Goli et al., 2014). They report that such a model allows determining the travel schedules of vehicles, the optimal route, and the number of required vehicles according to time windows defined for customers. Asamoah et al. (2021) investigate how supply chain responsiveness can affect the ability of companies to attract, satisfy and retain clients. They show that operations systems responsiveness and supplier network responsiveness lead to the logistics systems responsiveness of companies. They also display that operations systems responsiveness and logistics process responsiveness can improve customer development. Kazancoglu et al. (2022) study the resilience of a sustainable global supply chain to curb disruptions due to the advent of the Covid-19 pandemic. They clearly show the existence of the linkage between agility, flexibility, and supply chain responsiveness. They also report that supply chain agility and supply chain flexibility impact supply chain responsiveness.

### 2.1 Manufacturing firms performance and its relation with SCM

Manufacturing Firms’ performance (MFP) in the SCM literature refers to the indicators of a firm’s overall effectiveness; further, it refers to how well a firm fulfilled its market and financial goals (Sahoo, 2021), which includes two types as market share performance and financial performance. The first type includes market share, the growth of market share, and the growth of sales. Market share may be a key firm goal independent of profitability (Altunts et al., 2018). Manufacturing firms with high market share might be able to willingness gain more sales in markets; furthermore, might be able to have leadership in the long run to achieve high profits (Nabass and Abdallah, 2019). According to Chan et al. (2017), the improvement of customer satisfaction and loyalty will contribute to market share growth, which can be transformed into better firm financial performance. The latter type includes return on investment (ROI), growth in return on investment, the profit margin on sales, and overall competitive position, all of which measure profitability. For a long time, financial instruments were used as a tool for comparing and evaluating a firm’s behaviour, and any firm adopting SCM best practices (i.e. strategic supplier partnership, information sharing, customer service management, lean/agile production, green logistics Postponement, and TQM, etc.) should lead to enhancing firm performance (AL-Shboul et al., 2017). This indicates that performance is not just a result of all inputs of the firm, but it is a result of the firm’s objectives, achievements, and measures for success, which can provide important feedback to enable managers to reveal progress, enhance motivation, and communication, and diagnose problems. In addition, it provides information about how good the firm is for its activities, and to how much its customers are satisfied if processes are under control, and in which areas we need improvements. Thus, MFP for this study refers to the level of satisfaction by stakeholders and customers within the firm.

Some empirical studies found that advanced manufacturing performance systems affect operational performance not only firm performance (Ayoub and Abdallah, 2019). In operations management (OM) literature, there are three operational practices namely lean, total quality management (TQM), and SCM practices. These practices are widely adopted by most MFs in developed countries, and share common objectives consistent with achieving sustainable customer value and continues enhancements that are replicable across all departments within a firm and across its SCs as well (Sharma and Modgil, 2019). Meanwhile, SCM is a process of planning, organizing and controlling all activities within the SC to achieve cost-effective purchase of raw materials, components, items and services, while satisfying customer needs (Backstrand and Fredriksson, 2020). Additionally, SCM involves integration with other channel partners such as suppliers, intermediaries, third-party logistics providers, and even with competitors and customers as well (Kottala and Herbert, 2019). SCM has three main aims: cost reduction, capital reduction, and repair services (Seclen-Luna et al., 2021). Therefore, MFs and their SCs performance measurement have focused on three main elements: cost, time, and accuracy (Altuntas et al., 2018). Supply Chain Operations Reference (SCOR) is a model based-process and is defined as a process reference model, which is a widely used and accepted manufacturing industry reference model for all SC activities that assist MFs in mapping, developing, and referencing SC processes and operations and in assessing and monitoring levels of SC performance (Praharsi et al., 2022). This model of performance measurement features includes five main elements: reliability, responsiveness, flexibility/agility, cost, and assets (Pinheiro et al., 2020).

The literature indicates many studies have investigated and examined the relationships between MFP and SCM. For example, Asamoah et al. (2021) stated that the impact of exporting/growth in sales on productivity is higher for developing economies than for those that are developed due to the practice of integration with partners (Yang et al., 2019a, Yang et al., 2019b). explored the relationship between systems collaboration, strategic supplier partnership, SCR, and marker performance in manufacturing firms, and indicated that SCR significantly influenced the market share of MFP. The study of (Giannakis et al., 2019) emphasized on the relationship between information sharing, integration, SCR, TQM and market share performance. They proposed that SCR significantly influenced integration and market share performance, with their outcomes supporting the hypothesized positive impact. For instance (Jha and Sharma, 2022), explored the relationship between SCM practices, SCR (operations systems responsiveness, logistics process responsiveness, and supplier responsiveness), and competitive advantage (CA) and emphasized that SCR had a positive impact on CA of MFP (Giannakis et al., 2019). illustrate that advanced manufacturing systems affect operational performance not only firm performance. More specifically, manufacturing flexibility might play a role in lower inventories, reduced warehousing spaces, improve quality via faster feedback loops, and enhanced products and functions (AlHusain and Khorramshahgol, 2018). emphasized that manufacturing flexibility and responsiveness influencing positively operational performance, particularly in the elements of quality, speed, delivery, and elasticity; and pull production (Aljanabi and Ghafour, 2021). Surprisingly, the cost performance element was not influenced by manufacturing flexibility. The mediating role of SCR leads to export performance (Nenavani and Jain, 2022), financial performance (Andersson et al., 2019) and MFP (Qrunfleh and Tarafdar, 2013). (Asamoah et al., 2021) recognized the importance of flexibility in the SC process as antecedents to its agility and responsiveness in MFP. Additionally, they derive that manufacturing firms’ SC agility and responsiveness have been significantly influenced by the degree of flexibility in different processes such as procurement, manufacturing, and distribution (Yang et al., 2019a, Yang et al., 2019b). investigate the impact of manufacturing SCR on workflow and operational performance and find empirical and direct relation evidence that supports SCR on MFP (lower inventories and costs). Further, they found that manufacturing SCR also increases the speed to be in the market and the flow of materials and products (time). Therefore, manufacturing SCR is positively associated with MFP. The alignment between TLS and manufacturing SCR has gained considerable attention in a very few empirical studies. The development of TLS in alignment with manufacturing SCR can serve to enhance and tailor the product offering for customers as well as enhance the internal efficiency and effectiveness of MFP as well (Huge-Brodin et al., 2020). Therefore, TLS is a subset of SCM practices and MFP.

### 2.2 Transportation logistics strategy and supply chain responsiveness view

TLS refers to the achievement of the long-term objectives and goals of the firm and/or SC. Therefore, it requires the adoption and implementation of this term for the integration of operations and logistical activities in the firm's workforce and throughout the value-added chain in order to provide the optimum advantage to customers (Yokokawa et al., 2018). The transport sector plays an important role in the development and well-being of societies due to the interaction of this sector with various industrial and service sectors through rapid response to their supply chains to reach sustainability processes and provide the necessary products in different markets in various ways to achieve the satisfaction of its customers (Pinheiro et al., 2020). The transportation sector includes the movement of people and goods by cars, trucks, trains, ships, airplanes, and other vehicles. The high growth rates of transportation activity has pushed most MFs to focus on its strategy to be more flexible and responsiveness due to the volatile and unstable markets (Tsvetkova and Gammelgaard, 2018). Either by vans, by trailers, by train, by airplane, or by vessel; each means of transportation logistics has its constraints related to responsiveness; therefore, it needs a flexible and responsive strategy by several MFs to fit with rapid changes in customer requirements and needs due to numerous traffic problems such as severe traffic congestion and road accidents (Fulzele et al., 2019). There is an emerging consensus that transportation logistics system and its responsive strategy can mean the fast point-to-point transport available, or reliable and predictable journeys, or the quickest means to move products and goods freight, or journeys that use the least amount of time and cost or other resources to fulfil the customer orders (Diaz et al., 2022). This system for sure will offer attributes of effectiveness and impacts on economic development, and social quality of life.

Freight transport activities and its strategy are big challenges when it comes to improving SC sustainability and responsiveness. Hence, considering the increased highlights on sustainability and responsiveness within the SCM discipline, it is somewhat surprising that the focus on the transportation strategy and logistics activities are relatively scarce within the SCM literature (Erikson et al., 2022). However, many studies have highlighted the basic and complex embedding of different transport processes in supply chains and their responsiveness impact on its to customer requirements (Andersson et al., 2019). Therefore, there is a need to analyse the TLS as a part of wider logistics and SCM contexts. Dell highlighted the “Air to Sea Initiative” and the importance of aligning transportation strategies in the supply chain and the characteristics of transportation and industry operations Ke et al. (2015). Transportation creates time and place utilities and is considered a backbone of supply chain strategy. Transportation connects the operational activities between shippers and consignees across regions; therefore, the selection of transportation modes is correlated not only with logistics costs but also with customer satisfaction, financial cash flows, supply chain efficiency, and ultimately competitive advantage. Therefore, Eiriksson et al. (2022) state that the selection of transportation modes (intermodal) has become a strategically crucial decision in global SCM.

Applying and adopting an appropriate TLS at any MF have affected significantly the SCR network in any firm and in any country in terms of on-time delivery, fulfilling customer wants, inventory control, shorter lead times, speed, and efficiency. Firms that adopt effective TLS are aimed at removing and minimizing all wastes from their resources, and that will lead to creating efficiencies in their SC network through effectively managing logistical transportation and inventory, and improving the SCR process; thus, minimizing the wasting time (Sahoo, svetkova 2021 and Gammelgaard, 2018). Thus, it may obtain and apply the following issues: (1) mechanism of applying just in time through by delivering the required quantity of goods from the source of supply until reaching the manufacturing site directly on time and in good conditions with a low level of damages; and (2) may utilize various transportation logistical modes based on reliability and speed to achieve driven lowest cost strategy (Ning et al., 2020; Balajee et al., 2020). Thus, the aim of the adoption and application of SCR is to make the firm and its supply chain processes more flexible, efficient, and rapid to meet the changing demands of customers (Yang et al., 2019a,b; Shi et al., 2019). Thus, it permits the firms to produce customized products via their SC for their customers (Chen et al., 2018).

Many studies in the SC literature (Diaz et al., 2022; Qrunfleh and Taradar, 2013) have affirmed the correlation between the manufacturing firms’ SCR and MFP. Specifically, they suggest that TLS and SCR should improve and corporate the manufacturing firms’ reliability, speed, responsiveness, and performance in different locations and markets (Barrot et al., 2020; Banerjee et al., 2018).

### 2.3 Supply chain responsiveness, delivery reliability and delivery speed

Koh et al. (2007) define the responsiveness term as *“the firm’s ability to adapt to changes in its environment”* (Koh et al., 2007: 107); While it is defined by Juttner and Maklan (2011) as *“being able to bend easily without breaking”* (Juttner and Maklan, 2011: 247). Moreover, many studies have defined "velocity" and "speed" as a direct response to demand, and reaching it in the shortest period of time means performing functions and activities very quickly (Yang et al., 2019a, Yang et al., 2019b). Offering a variety of products, in high volume sized orders, with configured orders, and with handled orders, all these issues are defined under the flexibility response feature, which means that the firm can meet the unique requirements of customers in a cost-effective manner (Eriksson et al., 2022). In addition, flexibility, speed, and agility in transportation logistical strategy give the firm the opportunity to achieve success in its performance and expand its work by providing customized products in its warehouses in order to meet customer requests. Thus, building a bridge of trust and cooperation for strategic partnership rel with suppliers, external partners, and even consumers within the SC network will support and enhance the flexibility, speed, and responsiveness of the SC by creating better understanding between all partners (Ning et al., 2019a, Ning et al., 2019b). McCormack et al. (2008) state that “*in order to meet the performance levels demanded by today’s customers in terms of quantitative and qualitative flexibility of service in demand fulfilment, delivery consistency and reduction of lead times related to fulfilling orders, firms have developed repertoires of abilities and knowledge that are used in their organizational process”* (McCormack et al., 2008: 272). Thus, numerous manufacturing firms are currently focusing on using various types of transportation logistics modes as a crucial factor to respond quickly to the volatile signals of demands in a reliable way; so, this practice is an important element to attaining an integration among SC members (Praharsi ta., 2022; Ning et al., 2019a,b).

Delivery reliability is defined by many research studies as the range to which the firm is eligible and able to provide the demanded customer orders without delay with different kinds and sizes of products (Reardon et al., 2020). Xu (2017) states that there are four main elements of flexibility as follows: customer service, order, location, and delivery time. Whereas, other studies in the literature mention that there are various kinds of flexibility as volume flexibility, which refers to the ability to effectively increase/decrease total production in regards to responding effectively to customer demands (Inoue and Yasuyuki, 2020); delivery flexibility and speed: refers to the firm’s ability to adapt in shorter lead-times to the customer needs (Yokokawa et al., 2018); and finally, response flexibility: refers to the ease (in terms of cost, time, or both) with which the operation can be changed (Slack, 1991). Thus, delivery flexibility can be measured by the following dimensions: product flexibility (customization of products), volume flexibility, launch flexibility (introduce new products), access flexibility, delivery flexibility, and responsiveness to target markets flexibility (Yang et al., 2019a, Yang et al., 2019b; Banerjee et al., 2018).

In the relevant literature about the type of flexibility, research has provided inconsistent findings on the effect of manufacturing flexibility on firms’ performance indices such as market share, sales growth, and profits (Shi et al*.*, 2019). Therefore, through theoretical literature, some studies have indicated that many efforts have been made to improve the level of flexibility in manufacturing, transportation logistics, warehousing, and distribution. In some exceptional cases, the excessive increase in flexibility may presently rise up to negative findings (Barrot et al., 2020; Chen et al., 2018). Thus, some firms in some cases don’t get benefit from implementing favourable and proper internal flexibility for their operations in an uncertain environment; whereas, in some cases seems that more flexibility is not enough to achieve a high level of competitiveness. On the other hand, other studies confirmed the positive effect of flexibility on the firms’ performance, such as the study of Jha and Sharma (2022), who emphasized the significant impact of product mix and launching new product flexibility on increasing total revenues. In addition, companies have provided various products in order to increase their market share (Nenavani and Jain, 2022; Andersson et al., 2019); Whereas other research, such as the study of (Suarez et al., 1996) where the study found in its findings that there is a significant impact of volume flexibility on the total earnings and ability to compete.

### 2.4 The relationship between transportation logistics strategy and supply chain responsiveness, and the mediating role of delivery reliability, and delivery speed

**Figure 1:**
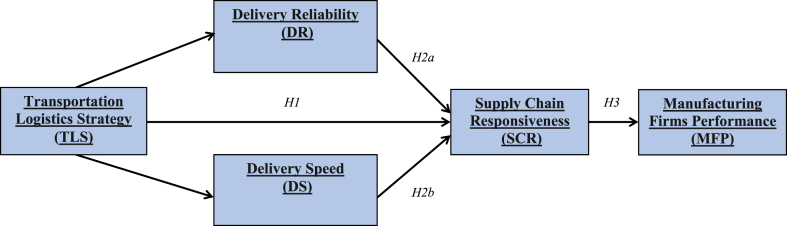
Conceptual research framework and hypotheses development.

Recent studies indicate that there is an effective linkage between firms and their SCs through their transportation logistics strategy to attain improvements in performance and optimal utilization of operations, functions, and activities (Jha et al., 2022; Praharsi et al., 2022; Yang et al., 2019a, Yang et al., 2019b; Altuntas et al., 2018). Diaz et al. (2022), Nenavani and Jain (2022) reveal that SC logistical activities including TLS, which is considered a crucial and effective element in the manufacturing firm’s ability to achieve outstanding execution in implementing a flexible SC strategy for all its internal and external operations. However, there is a shortcoming in the SC literature to a large extent in providing and offering proven theoretical or empirical frameworks in an investigation of such issues (Eriksson et al., 2022; Asamoah et al., 2021; Sahoo, 2021; Tsvetkova and Gammelgaard, 2018; AlHusain and Khorramshahgol, 2018; Aggarwal, 2018). In regards to the debates in this section, the researchers propose that there is a relationship between TLS and SCR, and might be mediated by the existence of suitable SC logistical transportation features (i.e., delivery reliability and delivery speed). Additionally, SCM literature did not specify and discuss which transportation logistics characteristics that when linked with TLS enhance and consequently improve the SCR. The conceptual model as illustrated in Figure 1 is an effort and contribution by researchers to bridge these two significant gaps in SC knowledge. The researchers propose that the characteristics of transportation logistics strategy mediate the relationship between TLS and SCR and that SCR is correlated with outstanding firm performance. More precisely, the researchers hypothesize that SC transportation logistics characteristics namely delivery reliability and delivery speed mediate the relationship between TLS and SCR.

## 3 Conceptual model and hypotheses development

The conceptual research framework for this research study is illustrated in Figure 1, which depicts the relationship between TLS and SCR and will be mediated by adequate characteristics transportation logistics strategy characteristics such as delivery reliability and delivery speed. In addition to that, the researchers propose that SCR is significantly positively related to improve MFP. All logical research hypotheses H1, H2a, H2b, and H3 are illustrated in Figure 1, which introduces and generates a schema for its relations.

### 3.1 The linkage between transportation logistics strategy and supply chain responsiveness

Prior research mentioned that there is a linkage between effective and appropriate characteristics of TLS, SCR, and firm performance (Heinen et al*.*, 2019). Therefore, the strategic choice of transportation logistics operations will have a significant and direct impact to support the SC in smoothing the flow of raw materials, products, goods, components, and items from upstream to downstream logistics. Additionally, it emphasizes reducing redundant costs by effectively managing the transportation logistics and inventory operations through SC processes. Thus, by minimizing inventory to the lowest levels and facilitating the soft flow of products via SC, the SC will have the potency to decrease and adjust the set-up times, capacity monitor, and react speedily to customers' orders. In short, an appropriate and effective TLS is foreseeable to improve the SCR for most MFs through SC resilience, shorter lead times, integrated SC processes, supplier performance, and customer responsiveness. Gunasekaran et al. (2008), In addition to that, the flexibility of the SC has several benefits such as, enhancing supplier performance, eliminating wastes from all resources, and being fast in the markets (Ning et al., 2019a,b; Feng et al., 2019) and increase the level of customer satisfaction and responsiveness (kanika and Shekhar, 2020). Thus, the researchers hypothesized in H1 that the adoption of an appropriate and effective TLS will improve and support SCR. This drives to formulating of the below hypothesis:

H1 Supply chain responsiveness is enhanced and supported by the availability and adoption of an appropriate and effective TLS.

### 3.2 The mediating roles of delivery logistics features (delivery reliability and delivery speed)

In the SCM literature, there are several types of research that highlight the role and significance of various logistical activities such as transportation and logistical distributions (i.e., delivery modes, etc.). Additionally, how they have a significant impact on these processes on enhancing the performance of firms, and how deliverables are used to attain and improve the performance through their SC logistical modes. Which, require internal integration across several functions within the firm as well as achieving incorporation with their providers and external partners, to attain more successful levels (Eriksson et al., 2022; Shi et al*.*, 2019). The majority of studies indicated that there is a relationship and linking directly logistical activities, including logistical transport operations, to either attaining a relative advantage, maintaining its sustainability, or improving manufacturing firm's performance (Reardon et al., 2020; Tsvetkova and Gammelgaard, 2018) or enhancing MFP without thinking about inserting an intermediary to obtain the findings of the different activities of SC (Barrot et al*.*, 2020; Giannakis et al., 2019). Based on what was mentioned in the background of the theoretical framework, the researchers suggest that the logistics activities (i.e. delivery dependability & reliability) mediate the relationship between TLS and SCR to reinforce MFP. Thus, researchers proposed in H2a that adopting an appropriate and effective TLS enhances and supports SCR through adopting and/or using high reliable delivery modes. For central firms, using up-to-date and reliable delivery modes will aid in reducing costs, minimizing levels of inventory, attaining shorter arrival times, achieving very few supply interruptions through different transportation logistics modes, and receipt of purchasing orders without delay, in the good quality (Hale et al*.*, 2020; Yang et al., 2019a,b). Adopting and using a high level of reliable delivery modes and offering proper effective TLS within the firm, will simplify the soft flow of required materials within the partners who are included within the SC network.

Thus, partners within the SC will be capable of understanding very well, foresee the focal firm, and know each partner needs by lowering uncertainty and enabling them to respond flexibly (Diaz et al., 2022; Qrunfleh and Tarafdar, 2013). The researchers propose in H2b that the availability of an appropriate and effective TLS enhances and supports SCR through adopting and/or using speedy delivery modes. This is will give the focal firm be more capable, reliable, and flexible to respond quickly to the changes in customer orders. In SCM literature, there are variety kinds of flexibility such as volume flexibility; processes flexibility; and delivery flexibility, which refers to the firm’s ability to adapt lead-times to the customer needs (Ning et al., 2019a, Ning et al., 2019b); range flexibility, and finally, response flexibility: refers to the ease (in terms of cost, time, or both) with which the operation can be changed (Slack, 1991). Additionally, several researches obtained “velocity” and “speed” into their flexibility definition and confirm that flexibility means doing different activities and processes in fast way (Ning et al., 2019a, Ning et al., 2019b). So, the researchers hypothesize that SCR is accomplished by including necessarily reliable and speed deliveries within a firm's TLS, and that the relationship between TLS and responsiveness is mediated by adopting and using highly reliable and speed delivery modes. Thus, H2 is formulated and hypothesized as follows:

H2a Delivery reliability mediates the relation between the availability of an appropriate and effective TLS and SCR.

H2b Delivery speed mediates the relation between the availability of an appropriate and an effective TLS and SCR.

### 3.3 Relationship between SCR and MFP

Many prior researches categorized performance measures into two kinds: market share and financial. Li et al. (2006) define firm performance as “*how well an organization achieves its market-oriented goals as well as its financial goals*”. Firms that have a high level of market share might be capable of increasing their sales growth from the markets; thus, they might be capable of being a leader in the long run to attain high profits (Chetty et al*.*, 2020). Enhancing SC performance for sure will lead to improving the firm performance as a whole (Chen et al*.*, 2018; AL-Shboul, 2017). Asamoah et al. (2021) state that firms with a high level of SC capabilities such as SCR, sharing of information, cooperation, and integration in all processes will have a potency and considerable significant and positive influence on enhancing a whole MFP. Whereas, manufacturing firms with high levels of reliability, flexibility and speed in their SC, for sure will be able to adapt in unstable and volatile demands in dynamic environments (Inoue et al*.*, 2020). The researchers hypothesize in H3 that SC with a high level of flexibility and agility will be able to respond quickly to detect the signals that are coming from the markets due to different and frequent changes will have a significant positive impact on MFP. Thus, it is hypothesized that:

H3 A supply chain with high level of responsiveness will have a significant positive effect on manufacturing firm performance.

## 4 Research methodology and findings

### 4.1 Measurement tool structure, content validity, reliability and pilot study

*Instrument tool structure*, A web-based survey was used to measure TLS main items, which is designed and formulated and developed by the questions contained in its content by the researchers; while the measurement tool was used to inspect SCR, delivery reliability, speed, and MFP was adopted from prior spacious studies that are related to this theme (Qrunfleh and Tarafdar, 2013; Green Jr. et al., 2008; Li et al*.*, 2006). Previous researches were examined and verified the previous constructs by the gathered data from manufacturing firms, which is consistent with the sample of this research. The main elements were used in the constructs of the study are to clarify the main content and define the constructs; further, some elements were used from other researches. The elements that are used to measure delivery reliability, and delivery speed constructs is adopted from studies like (Xu et al*.*, 2017; Green Jr. et al., 2008; Li et al*.*, 2006); whereas to measure SCR construct, the researchers adopted its elements from (Qrunfleh and Tarafdar, 2013) studies. Finally, the elements of MFP construct are adopted from prior studies as (Li et al*.*, 2006). All elements that are approved for this study were tested and measured by using a five-point Likert scale with choices representing 1 for strongly agree, and 5 for strongly disagree (AL-Shboul, 2017).

Quantitative research is adopted for this study. This type of research offers no intervention from the researchers or anyone else; only the respondents are responsible to fill the survey questionnaire (Patten, 2002). A web-based survey technique was used. It is more appropriate than other techniques (i.e. case studies, interviews, etc.) for data collection and analysis due to some limitations such as time and money. This tool was used because it represents an excellent technique for asking the respondents about their opinions, beliefs, and attitudes (Polland, 1998). Nevertheless, this study adopted a quantitative approach rather than a qualitative, one because the respondents in this study had highly demanding positions, so, they were too busy, and the researchers might not get the opportunity to interview them for the data collection process. Therefore, this method is considered easy for managers to fill out the survey freely because it will not take a lot of time from them. This tool has some advantages as it is suitable for statistical analysis, allows collecting a huge number of data from a large number of respondents, is versatile and standardized, economic, efficient, and generalizable (Hammersley, 1987). According to Cohen et al. (2000), state that web-survey is chosen because it has some advantages as follows:

• “Is economical, and efficient;
• Higher response rates;
• Fewer errors due to manual data entry;
• Represents a wide target population and is accessible from any device;
• Fast data collection, and generates numerical data for analysis;
• Provides descriptive, exploratory and inferential information;
• Manipulates key variables or factors to derive frequencies;
• Standardizes information gathered, and is flexible with research design;
• Enables data analysis to be performed (i.e. correlations)” (Cohen et al., 2000: 171).

Whereas, there are some key disadvantages of web-based surveys as emphasized by Gelder et al. (2010) as follows:

• “Concerns about the generality and validity of the results;
• Online Limitations, not everyone has internet access;
• Close-ended question limitations;
• Non-Response Bias;
• No interviewers, inflexibility and lack of potential depth;
• High chances of survey fraud, sampling Issues”

*Survey content validity*, to attain and verify a good level of validity of the questionnaire used in this study, four academics and three practitioners with long working experience, direct link, knowledge, and know-how in the domain of SC were asked to conduct a comprehensive review of the measuring instrument used in this research. Which includes five-point Likert scale to do the necessary and required adjustments to the redundant and/or ambiguous elements or delete them from the measurement survey tool, and thus adding new elements wherever it was crucial for the small-scale (pilot phase) study.

*Survey instrument reliability****,*** the major common and used measurement is Cronbach’s alpha test, which tests the internal consistency of the measuring instrument tool. Nunnally (1978) recommends and states that if alpha values have ≥0.7 will be accepted for all study elements.

*In the pilot study phase*, a small and limited empirical study sample was applied and conducted (pilot study) on forty-three responses out of two hundred and twelve (representing 20.3 percent response rate) distributed among targeted respondents similar to the large sample, who were randomly selected from the manufacturing companies sector to ensure proportional representation, which indicates that all sample is “given a known non zero chance of selection” in order to minimize the instrumentation threat as recommended by (Sekaran, 2000).

### 4.2 Method of data gathering and respondents' demographic information

*Targeted Population*, the population for this research consists of participants from the middle and upper management levels, which includes firm executives, vice presidents, supply managers, manufacturing managers, transportation and distribution logistics manager, planning manager, operations manager, information technology managers, and purchasing manager in various large manufacturing firms in Middle East territory. A private marketing company was dealt with to provide data and private emails to the study population, through which a web-survey was designed on the Internet for the process of collecting primary data^1^. A total 212 valid and completed responses out of 2,315, representing 9.1 percent response rate. Phone calls and e-mail messages have been made to the target participants in order to motivate and push them to fill out the survey and get guaranteed that it is filled out by them. Structural equation modelling (SEM) using AMOS software was performed for the analysis of completed surveys. Thus, non-response bias has been investigated, which is a significant origin of bias in survey research. Tests of homogeneity were applied on the participated respondents for early and late of targeted respondents' manufacturing firms’ years of work and service. The findings show that there are no significant differences between the early and the late respondents’ sets, this means that there was no significant bias, Table 2 illustrates all outcomes by performing Pearson’s chi-square test that were applied on two-waves of the targeted respondents (AL-Shboul, 2019). The common method bias was addressed by running Harman’s single-factor analysis test of the research elements to recognize the existence of popular method variance. The outcomes of this investigation technique test demonstrated that when covering all elements for all dimensions into exploratory factor analysis (EFA), it is found that there is not anyone factor that counts for a majority of the variances. The factor analysis method to determine common method bias was confirmed by a current research (Richardson et al., 2009).

Tables 3 and 4 show the respondents' profiles in the survey. As illustrated in Table 3, the majority of the respondents were junior and senior managers; While Table 4 illustrates that most of the respondents were from supplying/purchasing/logistics functions. Tables 5 and 6 explain general information for the participated manufacturing firms. Table 5 shows that most of the manufacturing firms surveyed include the food and textile sectors, taking into account that the percentages of all other sub-sectors were very close to each other, while the study indicated that most of the manufacturing firms have been in markets for at least ten years shown in Table 6.

### 4.3 Examining convergent, discriminant validity, and reliability of model’s constructs

The researchers performed Exploratory Factor Analysis (EFA) for every dimension in the model by using principal components as means of extraction, and VARIMAX as the method of rotation to enclose the unidimensionality of the scale. Table 1 shows all findings and measurement elements using factor loadings. Initially, twelve elements were included for TLS dimension. It has emerged only single factor structure from this analysis with three elements as follows: TLS4, TLS5, and TLS10. All these three elements are concerning directly to the delivery response and cost criteria. Therefore, the researchers deleted the three previous elements from any furthermore analysis. All remaining elements were loaded into their respective factors with 0.73 or more.

**Table 1:** Survey instrument and Factor Loading Analysis (FLA) findings for each construct.

Item	Factor loading from EFA	Factor loading from CFA	Reliability
***(a) TLS construct***			
TLS1 Evaluating frequently the fulfilment processes and distribution methods.	0.73	0.61	0.89
TLS2 Using efficient materials-handling equipment's in our delivery model(s).	0.78	0.69	0.91
TLS3 Managing inventory by delivering when and where we need.	0.77	0.62	0.86
TLS4∗ Responds quickly and effectively in most cases to our changing requirements of delivery time.	0.21	0.36	0.11
TLS5∗ Strives to coordinate inbound and outbound transportation and improve freight control through reducing an empty miles.	0.45	0.52	0.62
TLS6 Inspects all products frequently.	0.75	0.75	0.83
TLS7 Respond quickly and effectively to our changing requirements of cost.	0.87	0.83	0.86
TLS8 Can handle changes in several delivery mode(s).	0.79	0.72	0.81
TLS9 Responds quickly to customization products and/or orders.	0.81	0.81	0.84
TLS10∗ Offers higher-capacity delivery modes to respond to changes in the markets.	0.46	0.39	0.63
TLS11 Using IT tools in most logistics delivery modes for supporting communication and tracking.	0.79	0.67	0.88
TLS12 Updating frequently our logistics delivery mode(s).	0.83	0.75	0.79
***(b) SCR construct***			
SCR1∗ Our suppliers feed our firm a high level of quality of raw materials/items/components.	0.43	0.33	0.50
SCR2 Our SC is capable of offering customized products with required specifications for our customers.	0.79	0.78	0.77
SCR3∗ Our SC is capable of offering different characteristics of products such as colours, weights, sizes, and options.	0.54	0.61	0.55
SCR4 Our SC is capable to accelerate and/or decelerate production capacity responding to our customers’ orders.	0.84	0.80	0.92
SCR5∗ Our SC is capable to launch different kinds of product enhancements.	0.22	0.49	0.59
SCR6 Our SC is capable of lunch new products for customers.	0.87	0.79	0.78
SCR7 Our SC is capable of fulfilling customer demands without any delay.	0.71	0.66	0.84
SCR8 Our SC has terse order-to-delivery time.	0.77	0.76	0.79
SCR9∗ Our SC is capable to do and perform the required variations for different types of products.	0.66	0.48	0.56
SCR10 Our SC is fast in customer response time.	0.78	0.71	0.88
SCR11∗ Our SC has a visible system from points of supply until points of use.	0.29	0.58	0.65
SCR12 Our SC can achieve a high level of integration through the Information system that covers all functions and processes.	0.82	0.77	0.85
***(c) DR construct***			
DR1 Our firm is capable to ship different kinds of products in good conditions.	0.72	0.61	0.78
DR2 Our firm is capable to ship various customers’ requests without any delay.	0.73	0.69	0.89
DR3∗ Our firm is frequently updated delivery modes.	0.53	0.63	0.61
DR4 Our firm provide dependable delivery.	0.88	0.82	0.81
***(d) DS construct***			
DS1∗ Our firm is capable of shipping different types of products in an unstable markets.	0.47	0.57	0.55
DS2 Our firm is capable to ship customized orders for customers.	0.80	0.76	0.87
DS3 Deliveries in our firm are able to adjust/alter their routes based on the customer demand changes.	0.75	0.78	0.75
DF&S4∗ Our firm provide fast and high reliable delivery modes.	0.62	0.38	0.57
***(e) MFP construct***			
MFP1 Market share.	0.77	0.69	0.85
MFP2 Return on investment.	0.84	0.87	0.88
MFP3 The growth of market share.	0.88	0.80	0.90
MFP4 The growth of sales.	0.87	0.74	0.81
MFP5 Growth in return on investment.	0.82	0.78	0.79
MFP6 Profit margin on sales.	0.80	0.80	0.83
MFP7∗ Overall competitive position.	0.59	0.60	0.53

∗ Denote items were dropped.

**Table 2:** Illustrates all outcomes by performing Pearson’s chi-square test that were applied on two-waves of the targeted respondents.

	Pearson’s chi-square test	Value	df	Asymp. Sig. (2-taild)
Two waves test on targeted respondent firms job titles	Pearson chi-square	3.986^a^	5	0.028
	Likelihood ratio	5.431	5	0.254
	Linear-by-linear association	0.029	2	0.673
	N of valid cases	43	-	-
Two waves test on targeted respondents' firm job functions	Pearson chi-square	49.654^a^	29	0.023
	Likelihood ratio	40.121	29	0.661
	Linear-by-linear association	0.063	1	0.568
	N of valid cases	43	-	-
Two waves test on targeted respondents' firms’ years of working in business	Pearson chi-square	68.876^a^	67	0.037
	Likelihood ratio	80.651	67	0.365
	Linear-by-linear association	0.473	1	0.654
	N of valid cases	43	-	-

**Table 3:** The targeted respondent firms job titles.

Job Title	Number of manufacturing firms targeted in the study (n = 212)	Percentage (%)
Junior managers	155	73%
Senior managers	51	24%
Directors and top managers	6	3%

**Table 4:** The targeted respondents' firm job functions.

Job function∗	Number of manufacturing firms targeted in the study (n = 212)	Percentage (%)
Top-level-executives	12	5%
Logistician/Supplying/Purchasing	113	53%
Productions/Operations	10	4%
IT/Planning	3	2%
Transportation/Distribution	60	28%
Sales	4	2%

**Notes:** ∗ There was a targeted number of respondents working in more than one field/area in the manufacturing firm, as this sample represented 9 percent of the total sample 212.

**Table 5:** The targeted respondents’ industry type/sector firm.

Industry type/sector	Number of manufacturing firms targeted in the study (n = 212)	Percentage (%)
Electrical/IT Manufacturing	12	5%
Food and Beverage Processing	44	21%
Chemical/Cosmetics Production	24	11%
Metal Processing	16	8%
Pharmaceutical/Medical	23	11%
Textile/Clothes Manufacturing	32	15%
Paper/Tissues/Packing Processing	19	9%
Furniture/Wood Manufacturing	18	9%
Plastic/Rubber Processes	22	10%
Others types	3	1%

**Table 6:** The targeted respondents' firms’ years of working in business.

The targeted respondents' manufacturing firms’ years of work and service	Number of manufacturing firms targeted in the study (n = 212)	Percentage (%)
Less than 3 years	2	1%
Equal 3 or less than or equal 6 years	22	10%
Equal 7 or less than or equal 10 years	46	22%
More than 10 years	142	67%

Whereas, initially twelve elements were included for the SCR dimension. Also, it has emerged only single factor structure from this analysis with five main elements loaded into all other elements with loadings more than 0.71. Thus, the researchers deleted and excluded the five elements from any further analysis as follows: SCR1, SCR3, SCR5, SCR9, and SCR11. The five elements are SCR1, SCR3, SCR5, SCR9 and SCR11. The researchers not included the previous mentioned five main elements from any additional analysis.

For DR and DS dimensions, eight elements were initially presented. The findings generated a double factor structure that emerged from this analysis with three main elements as follows: DR3, DS1, and DS4. All three previous elements are related directly to the delivery time, flexibility, speed, and ability issues. The researchers deleted all the three previous elements from any additional analysis. All remaining elements were loaded into their respective factors with 0.72 or more. Finally, seven elements were included initially for the MFP construct. It has emerged only single factor structure from this analysis with single element loaded-MFP7 element has removed-into all other elements with loadings 0.77 or more. Cronbach alpha analysis was used to measure the reliability for each construct in this study. Table 1 summarizes Cronbach alpha values for all contracts that range from 0.75 up to 0.92 (Nunnally, 1978).

For checking convergent validity, reliability, and discriminant validity of the survey used as a measurement tool, the researchers performed Structural Equation Modelling (SEM) analysis by applying Confirmatory Factor Analysis (CFA) for all study dimensions by using AMOS software 7.0. The entity of convergent validity is confirmed if the fit parameters are consistent and illustrate as reasonable value. The researchers applied a combination of fit acceptable and consistent parameters for this study as follows: Goodness of Fit Index (GFI), Adjusted Goodness of Fit Index (AGFI), and Root Mean Square Residual (RMSR). Figure 1 shows AGFI modifies freedom degrees of the conceptual framework; whereas, GFI explains the relative rate of variance and covariance together clarified by the conceptual research framework. The GFI amount figure is considered acceptable if it falls in the range between 0.8 and 0.9 as recommended by Joreskog and Sorbom (1989); the GFI value in this study is pointed higher than 0.9. While RMSR value will be considered acceptable if it is below 0.05. In our model, it is fit as follows: χ2/df of 3.35; CFI = 0.91; AGFI = 0.91; RMSR = 0.04. All these values are considered acceptable due to falling within the acceptable ranges as recommended by Gaskin (2013). Table 1 illustrates the findings of the FLs from CFA and all FLs above 0.61.

Discriminant validity was tested applying Average Variance Extracted (AVE); Maximum Shared Squared Variance (MSSV) and Average Shared Squared Variance (ASSV). The purpose of applying this technique is to test and measure the independence of the item in each dimension. The findings as mentioned in Table 6 illustrates that all MSSV and ASSV amount figures are lower than the AVE amount figures for every single dimension, which confirms associate for the discriminant validity of all dimensions included in the conceptual research framework of the study (Gaskin, 2012). Furthermore, and as a double-check test of discriminant validity, the square root of the AVE for each construct is found greater than the correlation of each construct to all other constructs as shown in Table 7 (Chin, 1998).

**Table 7:** Summarizes of study findings of AVE, MSSV, and ASSV for all constructs by using SEM Method.

	AVE	MSSV	ASSV	TLS	DD&R	DF&S	SCR	MFP
TLS	0.65	0.23	0.06	0.76				
DD&R	0.73	0.28	0.07	0.41	0.76			
DF&S	0.57	0.41	0.14	0.33	0.53	0.68		
SCR	0.55	0.15	0.08	0.04	0.05	0.03	0.76	
MFP	0.76	0.33	0.05	0.53	0.42	0.30	0.31	0.79

### 4.4 Findings of hypothesis testing

**Figure 2:**
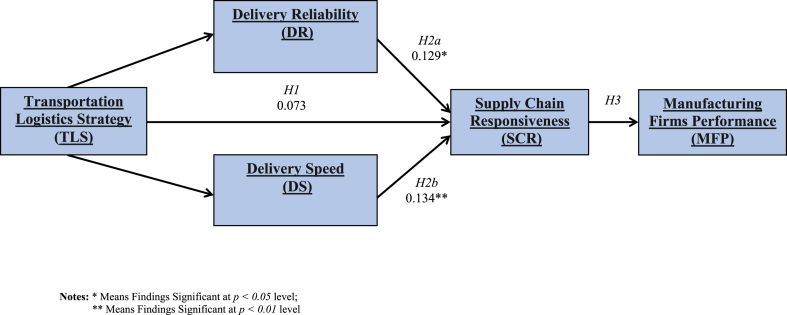
Path model with beta coefficients and significant confidence levels.

The findings of the study model applied by SEM and using AMOS, pointed out it has a favourable fit with a (χ2/df of 4.4, GFI = 0.957, AGFI = 0.881, and RMR at 0.05) as shown in Figure 2. The researchers conducted a bootstrapping test in order to check the indirect relationship for inserting a mediation relationship. This technique test is used to estimate and assess hypotheses Kenny (2011). In the first phase, it was examined all study hypotheses by running the direct impact of TLS items on SCR excluding any consideration of any mediating parameters. The estimation found 0.087 of the regression weight (β-value) between TLS and SCR relation; this value is greater than 0.05 level (two-tailed), which is considered not significant. This points out that there isn't an aligned significant impact in the relationship between TLS and SCR. Thus, H1 is not confirmed.

While, in the second phase, the researchers inserted the mediating parameters (i.e., DR and DS), then run out the model again with an application of 90 percent confidence level with 2000 bootstrapped samples.

In this scenario model, the researchers notified that there is a significant impact of TLS on SCR with the existence of the mediating parameters. Finally, the researchers also look at the nondirective impact of TLS on SCR by inserting the mediating parameters. The researchers emphasize the standardized lineal impact figure of TLS (0.059) on SCR as mentioned in the first case. Additionally, the researchers focus on the bootstrap confidence (bias-corrected percentile method) to be sure whether this value is enough significant or not. The findings illustrate that at the 0.05 level, there is no standardized significant effect between TLS and SCR relationship. While there is a nondirective impact of TLS on SCR with including of and presence of and the mediating parameters; the researchers focus on the standardized nondirective impact amount of TLS (0.152) on SCR parameter.

This value is considered worthy at 0.05 & 0.01 confidence levels. Table 8 summarizes the findings of β-coefficients and significance levels as shown in Figure 2. The researchers found that there is a significant impact of H2a hypothesis, this means that DR is partially mediates and have a significant effect on the relationship between TLS and SCR. Furthermore, it is found that DS is fully mediated the relationship between TLS and SCR, which means that H2b is also has a significant effect. Lastly, the estimation of the regression weight value is 0.311 between SCR and MFP; emphasizing that SCR has positively associated with MFP. Thus, the researchers reflect H3 is significant. The findings of all proposed hypotheses for this study is illustrated in Table 8.

**Table 8:** Summarizes of SEM analysis findings for all study dimensions.

Hypothesis	Direct β w/o Med	Direct β w/Med	Indirect β	t-values	Types of intermediate factors monitored	Sig. (P)
H1: TLS-SCR relationship	0.073	-	-	3.78	-	No
H2a: DR mediates TLS-SCR relationship	0.278∗∗	0.270∗∗	0.129∗	2.81	Mediates Partially	Yes
H2b: DS mediates TLS-SCR relationship	0.079	0.062	0.134∗∗	3.13	Mediates Fully	Yes
H3: SCR-MFP relationship	0.311∗∗	-	-	5.11	-	Yes

**Notes:** ∗ Means findings Significant at 0.05 level; ∗∗Means findings Significant at 0.01 level.

## 5 Discussion of findings and theoretical contributions

The researchers present some theoretical contributions for the knowledge of SCM. Firstly, it points out to emerging invitation for investigation how different logistics activities (i.e. characteristics of delivery logistics modes) support SCR (Qi et al*.*, 2011). Thus, the researchers propose a conceptual model for realizing how DR and DS as features and characteristics for transportation logistics delivery modes as parameters that mediate the TLS and SCR relationship. Furthermore, the researchers show that there is a partial mediation influence of DR on the relationship between TLS and SCR; whereas, DS is found fully mediation; and this leads to improve the SCs capability and enhances MFP. These relationships point out a contribution to the SCM literature that describes how TLS can boost SCR, through adopting, using and updating appropriately logistics delivery modes. Though the literature explains the concepts of DR and DS (Ayoub and Abdallah, 2019; Xu et al*.*, 2017; AL-Shboul, 2017; Li et al., 2006), by investigating DR and DS as mediating parameters in the relationship between TLS and SCR; the researchers contributed to focus on the DR and DS by exhibitionist their major and vital roles as likely means to suitable SCR from the respective of well-structured TLS items.

Secondly, the findings of H1 hypothesis in this research show that TLS did not has any significant effect to support SC. Thirdly, the findings indicate that there is no significant immediate effect in the LS and SCR relationship, Thus, this steers the researchers to deduce that the manufacturing firms focus only on the elimination of wasting resources without concerning the use of proper resources such as specific features of transportation logistics delivery modes, and this will attain SC benefits in terms of responsiveness. Therefore, if an appropriate, and effective TLS is available in the manufacturing firm with adopting and using specific high reliable delivery modes, this will find a pathway to SCR through using such modes. Such as DR can offer and provide the type of goods required in good conditions, shipping different demanded orders without delay, frequent update the types and capacity of their delivery modes; at the end will lead to enhanced SCR, which presents the significance of the hypothesis H2a*.* Fourthly, the findings of hypothesis H2b present that DS is fully mediating the relationship between TLS and SCR. Thus, it will enhance the relationship between TLS and SCR if it has a high level of different and/or several DS modes; further, it contributes and has an indirect boosting positive effect between TLS and SCR relationship. Additionally, any firm adopting, having different, reliable, and updated delivery modes will contribute a high level of responsiveness to their SCs. Further, any firm with a high level of flexibility, speed, leads to better understanding and quickly reaches the markets and satisfies their customers’ needs; thus, enhancing SCR. The literature on SCM has worthy studies, bears, and mentions the importance of SCR as Gunasekaran et al. (2008) study. While, it did not indicate and did not consider the critical and vital role of DR and DS in improving SCR. Fifthly, the findings of this study point out that any manufacturing firm with a high level of SCR will improve and attain high market share and financial performances in manufacturing firms as indicated in the H3 hypothesis. For example, the manufacturing firm will be more effective and responsive, flexible, and dynamic to volatile demanded changes in the markets. If their SCs are capable to react quickly, then the firm will become more agile and will achieve and sustain a valuable competitive advantage; thus, leads to improving customer services and MFP; since the intensified competition now is not only between manufacturing firms but also between SC networks (Ayoub and Abdallah, 2019; AL-Shboul, 2017; Li et al., 2006). Indeed, as illustrated in hypothesis H3 findings, it emphasizes SCR as a crucial element for better success of manufacturing firms by associating SCR with MFP. Lastly, this research illustrates the significant value of using appropriate and adopting an effective TLS at any firm for achieving and enhancing SCR. Especially, if industrial firms are looking to improve SCR, they must adopt, implement and use a number of different methods of reliable, flexible, and rapid transportation logistics modes to achieve a high level of responsiveness through their SCs. Based on the foregoing, the researchers emphasize the great significant roles of adopting, adapting, and using different and agile transportation logistics modes, and patterns in associating and improving SCR. To further explore the mediating relationship of reliable and fast transportation logistics modes such as DR and DS on the relationship between SCR and TLS, the researchers tested (i.e., post-hoc test) the mediation impact of DR and DS on the items of the relationship between TLS and SCR. The researchers conducted bootstrapping way, both standardized-direct and indirect impacts of TLS on SCR items with the presence of DR and DS are found significantly at *p* < 0.05. These findings point out that DR & DS as intermediate parameters are vital elements that can reinforce the TLS and SCR relationship for manufacturing firms. One interpretation of these findings could be that by offering, adapting, developing, and improving different criteria and characteristics within TLS by the manufacturing firm, the firm will have more opportunities to be more agile, flexible, and fast to markets to deliver the required orders of their customers’ orders by adopting, implementing, and using several high reliable and speedy transportation logistics modes that can enhance the responsiveness through their SCs.

## 6 Research implications, limitations, propose directions for the coming research, and conclusion

### 6.1 Research implications

Due to the shortage of studies in SCM literature indicating that diverse features and characteristics of transportation logistics delivery modes intermediate the TLS and SCR relationship, so this empirical research came to highlight a collection and combination of reliable measures for the examination and evaluation of TLS. The measurements that were developed for applying this empirical research focuses on and capture different aspects such as delivery time, reliable delivery, delivery capacity and capability, delivery flexibility, delivery responsiveness, delivery speed, etc. of the TLS dimension, and thus can be concerned a benchmark for other factors. Scholars, academics, and practitioners either to evaluate the TLS items or to understand the effect of TLS items directly on SCR, and indirectly on overall MFP can also use these different scales. From the managers' point of view, this study demonstrates the cause careful sight that should be implemented when deciding which transportation logistics modes should be used and adopted currently and in the long run to cope with the fast development and improvement of the transportation sector used by manufacturing firms and so that it should be consistent with their TLS. The manufacturing firms that are capable effectively to managing their different transportation logistics operations will lead to achieving considerable avails by enhancing their outward processes, reductions in costs and wastes, and being more resilience and flexible, responsive, and speed in receiving orders from customers as well, and overall earning potential competitive advantage. Additionally, this research emphasizes that TLS and SCR relationship cannot be applied in isolation at manufacturing firms alone (Aggarwal, 2018; Qrunfleh and Tarafdar, 2013). Directors and practitioners have to recognize the crucial role of transportation logistics delivery logistics factors and their features in the TLS and SCR relationship; thus, for manufacturing firms that are looking for efficient operations through their different features of delivery modes, they should consider which different types of transportation logistics modes-if possible-to be selected, adopted, used, and updated.

### 6.2 Practical implications

Overall, the empirical results can provide insightful implications for practitioners and managers. Companies tend to increasingly adopt supply chain management practices with the aim of better-producing supply chain responsiveness and having a more competitive advantage given that business competition seems to evolve not only among companies but also between supply chain partners. Afterward, the findings show that delivery reliability along with the delivery speed can improve supply chain responsiveness and an effective way to better implement supply chain management and therefore strongly maintain the competitive advantage of the company. The findings can show to managers and practitioners that firms are in need of higher responsiveness in order to fastly meet client needs as well as real-time visibility tools to raise operations and transport abilities planning to reduce losses related to supply chain disruption. As a result, companies can highly improve their firm performance ad in particular financial performance by reducing operational costs.

### 6.3 Limitations and propose directions for future research

Despite the real and tangible contributions to this empirical study on both the theoretical and practical levels, it encountered some restrictions during its application as follows:

Firstly, this study relied on only one type of primary data collection by means of a web survey. The researcher was not able to conduct interviews with the respondents due to time and money constraints. Secondly, the researchers used only one participant from each manufacturing firm to fill out the survey. Therefore, using only one participant for data collection rather than many is considered not preciseness way to demonstrate the TLS items, logistics delivery modes, SCR, and MFP; this may cause some inaccuracies in measurement; In addition, there is no full-proof way to ascertain whether the survey was filled out by the intended respondents or by others. Therefore, the researchers recommend applying various data gathering methods to attain more specific and adequate data such as triangulation (i.e. survey and conducting interviews) for future research; in addition, using several participants from each participating firm in a similar study considers an effort to improve the reliability of the research outcomes.

### 6.4 Conclusion

Despite the insufficient published research on TLS and its various effects on MFS in the SCM literature. This empirical study contributed to providing some guidance on how to adapt and adopt reliable logistic transport patterns and processes such as DR & DS, and how to use and apply them effectively, which contributed to the improvement and development of the system modern, fast and flexible logistics transportation modes by providing competitive advantages and enhancing them to obtain the SCR feature. Furthermore, this research demonstrates the vital role and mediating factors effect of two delivery logistics modes-DR and DS-on the respective relationship of TLS and SCR. Going with this scenario, describes a theoretical sight of the crucial role between TLS items and delivery logistics characteristics modes, as a means to improve SCR and MFP as well.

## Declarations

### Author contribution statement

All authors listed have significantly contributed to the development and the writing of this article.

### Funding statement

This research did not receive any specific grant from funding agencies in the public, commercial, or not-for-profit sectors.

### Data availability statement

The data that has been used is confidential.

### Declaration of interest’s statement

The authors declare no conflict of interest.

### Additional information

No additional information is available for this paper.

## Appendix A Supplementary data

Questionnaire Questionnaire

## Footnotes

1 The questionnaire-based survey is very useful to collect data in this study in order to examine the impact of delivery reliability and delivery speed on the relationship between a manufacturing firm's transportation logistics strategy and supply chain responsiveness. It is very important to note the lack of direct measures to compute concepts under consideration. In fact, many researchers have extensively used questionnaires in studying supply chain responsiveness in different (Nenavani and Jain, 2022; Praharsi et al., 2022; Eriksson et al., 2022; Asamoah et al., 2021; Sahoo, 2021; Giannakis et al., 2019; Ayoub and Abdallah, 2019; Yang et al., 2019a, Yang et al., 2019b; AlHusain and Khorramshahgol, 2018).
