# Supplementary material for: An investigation of transportation logistics strategy on manufacturing supply chain responsiveness in developing countries: the mediating role of delivery reliability and delivery speed
Source: Heliyon. 2022 Oct 27;8(11):e11283. doi: 10.1016/j.heliyon.2022.e11283 (PMC9647434; doi:10.1016/j.heliyon.2022.e11283)
Supplement: Questionnaire [file mmc1.docx]

**Questionnaire**

**Table 1.** The instruments and factor loading analysis findings for each construct

| **Item** | **Factor loading from EFA** | **Factor loading from CFA** | **Reliability** |
| --- | --- | --- | --- |
| 1. ***TLS construct***   TLS1 Evaluating frequently the fulfilment processes and distribution methods.  TLS2 Using efficient materials-handling equipment's in our delivery model(s).  TLS3 Managing inventory by delivering when and where we need.  TLS4* Responds quickly and effectively in most cases to our changing requirements of delivery time.  TLS5* Strives to coordinate inbound and outbound transportation and improve freight control through reducing an empty miles.  TLS6 Inspects all products frequently.  TLS7 Respond quickly and effectively to our changing requirements of cost.  TLS8 Can handle changes in several delivery mode(s).  TLS9 Responds quickly to customization products and/or orders.  TLS10* Offers higher-capacity delivery modes to respond to changes in the markets.  TLS11 Using IT tools in most logistics delivery modes for supporting communication and tracking.  TLS12 Updating frequently our logistics delivery mode(s).   1. ***SCR construct***   SCR1* Our suppliers feed our firm a high level of quality of raw materials/items/components.  SCR2 Our SC is capable of offering customized products with required specifications for our customers.  SCR3* Our SC is capable of offering different characteristics of products such as colours, weights, sizes, and options.  SCR4 Our SC is capable to accelerate and/or decelerate production capacity responding to our customers’ orders.  SCR5* Our SC is capable to launch different kinds of product enhancements.  SCR6 Our SC is capable of lunch new products for customers.  SCR7 Our SC is capable of fulfilling customer demands without any delay.  SCR8 Our SC has terse order-to-delivery time.  SCR9* Our SC is capable to do and perform the required variations for different types of products.  SCR10 Our SC is fast in customer response time.  SCR11* Our SC has a visible system from points of supply until points of use  SCR12 Our SC can achieve a high level of integration through the Information system that covers all functions and processes.   1. ***DR construct***   DR1 Our firm is capable to ship different kinds of products in good conditions.  DR2 Our firm is capable to ship various customers’ requests without any delay.  DR3* Our firm is frequently updated delivery modes.  DR4 Our firm provide dependable delivery.   1. ***DS construct***   DS1* Our firm is capable of shipping different types of products in an unstable markets.  DS2 Our firm is capable to ship customized orders for customers.  DS3 Deliveries in our firm are able to adjust/alter their routes based on the customer demand changes.  DF&S4* Our firm provide fast and high reliable delivery modes.   1. ***MFP construct***   MFP1 Market share.  MFP2 Return on investment.  MFP3 The growth of market share.  MFP4 The growth of sales.  MFP5 Growth in return on investment.  MFP6 Profit margin on sales.  MFP7* Overall competitive position. | 0.73  0.78  0.77  0.21  0.45  0.75  0.87  0.79  0.81  0.46  0.79  0.83  0.43  0.79  0.54  0.84  0.22  0.87  0.71  0.77  0.66  0.78  0.29  0.82  0.72  0.73  0.53  0.88  0.47  0.80  0.75  0.62  0.77  0.84  0.88  0.87  0.82  0.80  0.59 | 0.61  0.69  0.62  0.36  0.52  0.75  0.83  0.72  0.81  0.39  0.67  0.75  0.33  0.785  0.61  0.80  0.49  0.79  0.66  0.76  0.48  0.71  0.58  0.77  0.61  0.69  0.63  0.82  0.57  0.76  0.78  0.38  0.69  0.87  0.80  0.74  0.78  0.80  0.60 | 0.89  0.91  0.86  0.11  0.62  0.83  0.86  0.81  0.84  0.63  0.88  0.79  0.50  0.77  0.55  0.92  0.59  0.78  0.84  0.79  0.56  0.88  0.65  0.85  0.78  0.89  0.61  0.81  0.55  0.87  0.75  0.57  0.85  0.88  0.90  0.81  0.79  0.83  0.53 |

*Denote items were dropped.
